# Generic Pheromones Identified from Northern Hemisphere Cerambycidae (Coleoptera) Are Attractive to Native Longhorn Beetles from Central-Southern Chile

**DOI:** 10.3390/insects13111067

**Published:** 2022-11-18

**Authors:** Tomislav Curkovic, Diego Arraztio, Amanda Huerta, Ramón Rebolledo, Arly Cheuquel, Américo Contreras, Jocelyn G. Millar

**Affiliations:** 1 Facultad de Cs. Agronómicas, Universidad de Chile, Santiago P.O. Box 1004, Chile; 2 Facultad de Cs. Forestales y de la Conservación de la Naturaleza, Universidad de Chile, Santiago P.O. Box 9206, Chile; 3 Facultad de Cs. Agrícolas y Recursos Naturales, Universidad de La Frontera, Temuco P.O. Box 54-D, Chile; 4 Department of Entomology, University of California, Riverside, CA 92506, USA

**Keywords:** (2R*,3S*)-2,3-hexanediol, (2R*,3R*)-2,3-hexanediol, 3-hydroxy-2-hexanone, *Calydon submetallicum*, Chenoderus testaceus, Eryphus laetus, intercept traps

## Abstract

**Simple Summary:**

In the family Cerambycidae, most attractant pheromones identified to date have come from species native to the northern hemisphere. Because many of the pheromone compounds are shared among related species, field tests of known pheromones in new regions have frequently attracted additional species whose pheromones have not yet been formally identified. Here, we report the results of field bioassays with previously identified cerambycid pheromones in Chile, where pheromones have not been identified for any native cerambycids to date. Trials were conducted in several different localities in central-southern Chile, testing eight compounds individually. Approximately 580 specimens were captured from eleven species, with *Calydon submetallicum* showing significant attraction only to 3-hydroxy-2-hexanone, whereas *Eryphus laetus* was significantly attracted to this compound, as well as to (2R*,3S*)-, and (2R*,3R*)-2,3-hexanediol. These compounds are likely aggregation pheromone compounds for these species, and can be exploited for monitoring and biological studies of the Chilean cerambycid fauna.

**Abstract:**

We conducted field bioassays with several known cerambycid pheromones in two zones of central-southern Chile: (1) Las Trancas (Ñuble region) and Coñaripe (Los Rios region) (Study 1) and (2) Rucamanque and Maquehue (La Araucania region) (Study 2). Up to eight compounds were tested individually, including 3-hydroxy-2-hexanone, (2R*,3S*)- and (2R*,3R*)-2,3-hexanediol, fuscumol, fuscumol acetate, monochamol, 2-methylbutanol, and geranylacetone. Compounds were loaded in plastic sachets placed in either multiple funnel or cross-vane panel traps hung in trees in a randomized block design (*n* = 3 or 4). The number of treatments and bioassay periods varied depending on the study. A total of 578 specimens belonging to 11 native species were collected, with the three captured in the highest numbers being *Eryphus laetus* (292 specimens), *Calydon submetallicum* (*n* = 234), and *Chenoderus testaceus* (*n* = 20). The three species are of economic importance: *E. laetus* is considered a minor pest in apple orchards, and the other two species infest *Nothophagus* hosts, including some timber species. Traps baited with 3-hydroxy-2-hexanone collected significant numbers of both sexes of the two most abundant species, and this compound was the only treatment that attracted *C. submetallicum*. (2R*,3R*)- and (2R*,3S*)-2,3-Hexanediols were also significantly attractive to *E. laetus*. Our results suggested that 3-hydroxy-2-hexanone and 2,3-hexanediols, which are known pheromone components of cerambycid species worldwide, are also likely to be conserved aggregation pheromone components among some species in western South America.

## 1. Introduction

Longhorn beetles (Coleoptera: Cerambycidae) comprise a large and diverse family of insects, which are both ecologically and economically important. There are more than 36,000 species described in eight subfamilies worldwide [1]. The larvae are xylophagous or saproxylic, and generally perform valuable ecosystem services by initiating degradation and nutrient recycling of woody tissues in forests [2]. However, a few species have become economic pests, damaging crops, fruit orchards, or timber [1]. The larvae may have development times of several years, whereas the adult stage is much shorter, and is focused mainly on reproduction and oviposition. Mate finding and recognition is mediated by semiochemical signals and cues [3]. Even though there has been evidence for their use of semiochemicals since the 1960s [4], the first pheromones of cerambycids were not identified until the 1980s [5,6], and pheromones had been identified from fewer than 10 species up until 2004 [7]. However, in the past two decades, there has been rapid progress in cerambycid chemical ecology, with pheromones or likely pheromones now known from several hundred species, mostly from the northern hemisphere [8,9]. The cumulative data also show that there appear to be two types of attractant pheromones, with species in the subfamilies Lamiinae, Spondylidinae, and Cerambycinae using male-produced aggregation-sex pheromones which attract both sexes (with one exception [10]), whereas species in the subfamilies Prioninae and Lepturinae appear to use female-produced sex pheromones that attract only males [11,12]. The data also indicate that several pheromone compounds are widely conserved among related taxa, with compounds such as 3-hydroxy-2-hexanone being shown to attract species in the subfamily Cerambycinae on six continents. For some of these species, the attractive compounds have been shown to be produced by the responding species, whereas other species have been attracted to tested compounds, but it is not yet known whether these species actually produce the compounds to which they are attracted. Thus, it has often been the case that during field trials targeting a particular species, one or more related species have also been attracted, providing leads to the likely pheromones of the latter groups of species [13,14,15].

Over the past decade, some work has been carried out on the pheromones of cerambycids from eastern South America (e.g., Brazil [16], Uruguay [17]), but almost nothing is known about the chemical ecology of the cerambycid fauna native to countries in the western part of the continent, except for a recent paper from a locality (east of the Andes) in the Amazon rain forest of Peru [18]. In the Mediterranean habitats in the Pacific southwest of the subcontinent, there is also evidence for a female-produced sex pheromone in the species *Callisphyris apicicornis* (subfamily Necydalinae) [19,20]. In Chile, there are ca. 180 described cerambycid species [21], most of which are native, including some economically important ones. Thus, if some of the known and widely conserved pheromones which have been identified from species in other parts of the world also proved to be attractive to species native to western South America, they could serve as valuable tools for basic and applied studies for such species [22,23,24,25,26]. Therefore, the objective of the work reported here was to test a variety of known cerambycid pheromones as possible attractants for species on the southwestern side of the Andes for future development of monitoring tools for cerambycid pests, but also for biological studies of the Chilean fauna.

## 2. Materials and Methods

### 2.1. Studies, Localities, Seasons, and Site Characteristics

Study 1 was conducted in 2011 in localities in the Ñuble (Las Trancas: −36.912901, −71.504058; starting on 5 February), and Los Rios (Coñaripe: −39.586362, −72.021034; starting on 16 February) regions, where Patagonian oak saplings were the dominant trees. Traps were checked 5 days after they were deployed, in both localities.

Study 2 was conducted in 2018–2019 in two localities in the La Araucania region. The first site, at Rucamanque (−38.659444, −72.605611), was in an ecological reserve, dominated by vegetation typical of the Chilean original cold rain forest called “Bosque de la Frontera” [27]. The second site, at Maquehue (−38.836431, −72.694103), was dominated by Patagonian oak seedlings. Study 2 was initiated on 8 January, and trap captures were recorded weekly for 56 d in both localities. All sites belong to the central-southern zone of Chile, biogeographically mainly in the Maule province [28].

### 2.2. Traps, Compounds, and Treatments

The trapping protocols used in our field trials followed methodologies from similar studies conducted in other countries [12,15,26,29,30]. Lindgren funnel traps with two cones (Plasticos Los Cerrillos Ltd., Santiago, Chile) were used in 2011, while black cross-vane panel traps (AlphaScents Inc., Canby, OR, USA) were used in 2019 based on reports that these traps, when coated with a Teflon emulsion (tradename fluon^®^), increased capture efficiency [31]. Both types included a 1 l collecting cup at the bottom, filled with neutral detergent solution, for insect conservation until trap servicing. Traps were placed 10–20 m apart (we believe the distribution and distance between traps mostly avoided plumes overlapping) and hung on trees at 1.5–2.5 m height. Six (Study 1) or eight (Study 2) compounds reported as cerambycid aggregation-sex pheromones were tested individually. 3-Hydroxy-2-hexanone (common name 3-OH-6-2-Kt [6]: reference describing the first identification of the compound from a cerambycid species]), 2-(undecyloxy)ethanol (monochamol [32]), (*E*)-6,10-dimethyl-5,9- undecadien-2-ol (fuscumol [33]), (*E*)-6,10-dimethyl-5,9-undecadien-2-yl acetate (fuscumol acetate [16]), and 6,10-dimethyl-5,9- undecadien-2-one (geranylacetone [33], compound not available for Study 1) were purchased from Bedoukian Research (Danbury, CT, USA); 2-methylbutanol [34], (compound not available for Study 1) was purchased from Sigma-Aldrich (St. Louis, MO, USA), and (2R*,3S*)-2,3-hexanediol (R*S*-diol [35]) and (2R*,3R*)-2,3-hexanediol (R*R*-diol [6]) were synthesized as previously described [35]. All compounds were >95% pure by GC, except 3-OH-6-2-Kt, which was ~90% pure, with the major impurity being its degradation product, 2,3-hexanedione. Stock solutions were prepared by dissolving each compound in either ethanol (Study 1) or isopropyl alcohol (Study 2; the carrier solvent was changed because it is known that some cerambycids are attracted to ethanol, or ethanol has been shown to enhance attraction to pheromones [36]), and were kept in the freezer until use. Immediately before traps were deployed, either 0.5 mL of solution for Study 1 (doses were 25 mg monochamol, 50 mg 3-OH-6-2-Kt, R*S*- and R*R*-diol, and 100 mg fuscumol and fuscumol acetate) or 1 mL for Study 2 (50 mg doses for all compounds) were pipetted into Bagette plastic zip-lock bags (#14770, polyethylene, 5 × 10 cm and 0.05 mm wall thickness, Cousin Corp., Largo, FL, USA), serving as slow-release dispensers that were changed weekly in Study 2 in order to ensure good release rates, based on data showing release rates of some of the pheromone compounds from the zip-lock bags of several milligrams per day at 20 °C [29]. Thus, changing the lures weekly in Study 2 or testing the compounds only for 5 days in Study 1 was likely conservative. This is because the dispensers should emit compounds for at least one week for any doses tested, considering that the average temperature in trial sites was below 18 °C in January and February, and even lower in March [37]. Each bag was placed either in the first cone of funnel traps or in the center of panel traps. Baggies loaded with ethanol (Study 1) or isopropyl alcohol (Study 2) served as controls. 

### 2.3. Experimental Design, Results, and Statistical Analysis

A completely randomized block design (*n* = 3, Study 1, or *n* = 4, Study 2) was used in the two studies. Each block consisted of either 7 (Study 1) or 9 (Study 2) traps (6–8 treatments +1 control). A trap per treatment/block was the experimental unit. Trap counts were conducted once at Las Trancas and Coñaripe, 5 d after trap deployment, and for 56 d in the La Araucania localities. Beetles were identified by comparison with specimens from the Entomological Museum, University of Chile; specimens were sexed only for Study 2 based on antennae length and genitalia, for the three most common species. Results are presented as the mean captures/day (±SEM) and total catches (Table 1, Table 2 and Table 3). Data analysis was conducted by study and species, comparing all treatments, when total captures/study per species were >15 and all blocks had captures. Data were analyzed using the Friedman nonparametric test [38]. Differences between treatments were determined using the rank means, as described in [39]. Contrasts between sexes within a species were conducted only in Study 2. P- and T^2^- (Hotelling’s T-square) values are provided in tables. A 5% significance level was used in both tests.

## 3. Results

### 3.1. Study 1

Twenty-three *Eryphus laetus* Bl. (Cerambycinae [40]) were captured (Table 1), mostly in traps baited with 3-OH-6-2-Kt (*n* = 12) and R*S*-diol (*n* = 10 specimens), but no contrasts were performed, due to the absence of captures in any treatment in two blocks. Sixteen *Calydon submetallicum* Bl. (Cerambycinae) were also captured, all in traps baited with 3-OH-6-2-Kt, significantly different from the control and all other treatments.

### 3.2. Study 2

The number of species and the total number of specimens of each species caught in the second study are shown in Table 2. A total of 538 specimens were captured over 56 d of trap deployment. Most individuals (>96%) belonged to the subfamily Cerambycinae, including *E. laetus* and *C. submetallicum*, but also *Chenoderus testaceus* (Bl.), *Cotyachryson sulcicorne* (Germ.), *Maripanus quadrimaculatus* (Germ.), *Xenocompsa semipolita* (Fairm. and Germ.), and *X. flavonitida* (Fairm. and Germ.). We also captured *Aconopterus cristatipennis* Bl. and *Neohebestola humeralis* Bl. (both Lamiinae [41]), and *Hephaestion corralensis* Phil. and *Sternorhopalus virescens* (Fairm. and Germ.) (both Necydalinae [42]).

Two species were caught in significant numbers compared to the controls: *E. laetus* (*n* = 269) and *C. submetallicum* (*n* = 218). Catches of a third species, *C. testaceus* (*n* = 20) were marginally significant overall (*p* = 0.065), but there were no significant differences among treatments. Eight other species were captured in low numbers. Considering total catches, most cerambycids were captured in traps baited with 3-OH-6-2-Kt (61%), R*S*-diol (11%), and R*R*-diol (7%). *Eryphus laetus* specimens were captured in all treatments, whereas *C. submetallicum* were captured almost entirely in traps baited with 3-OH-6-2-Kt. Statistical analysis for *E. laetus* total catches showed significant differences between 3-OH-6-2-Kt, R*S*-diol, R*R*-diol, monochamol, and fuscumol acetate from the control, whereas only the first compound was different from the control in the case of *C. submetallicum*. No contrasts were conducted for the remaining species, given the low levels of captures.

**Figure 1:**
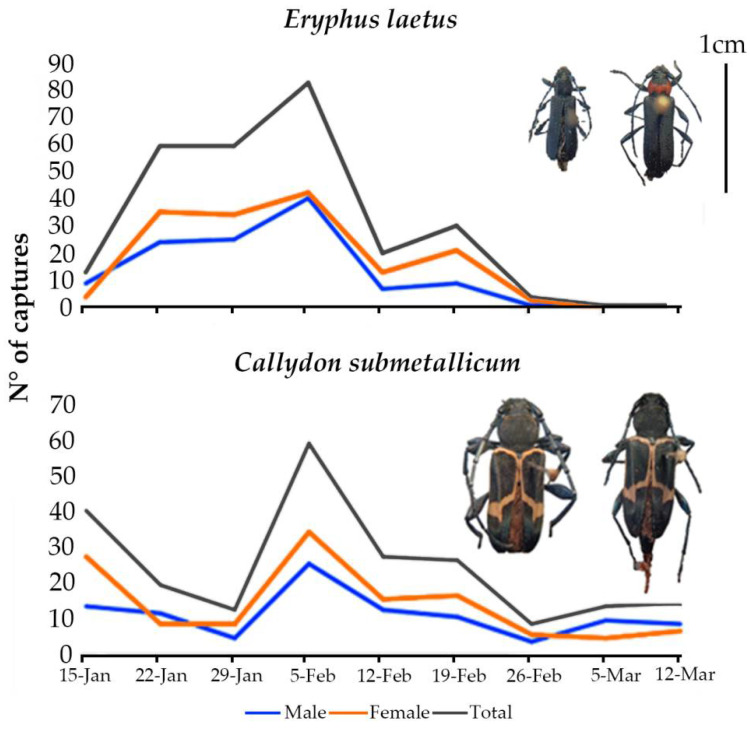
*Eryphus laetus* and *C. submetallicum* captures by date, from all treatments during Study 2, La Araucania region, 2019. Photographs show the males (left) and the females (right) for each species. Sizes at relative scale between specimens: a 1 cm black bar is displayed as reference.

Regarding the timing of catches, *E. laetus* and *C. submetallicum* were caught throughout the monitoring period, from January to March (Figure 1), showing peaks during the second half of January and the beginning of February, respectively.

Despite *E. laetus* overall catches being slightly female-biased (153:116), catches with 3-OH-6-2-Kt were male-biased, whereas those with the two diols were female-biased (Table 3). All other treatments were not statistically different from the control, except for fuscumol acetate in males. In the case of *C. submetallicum*, catches were again female-biased (123:95), with 3-OH-6-2-Kt being different from all other treatments for both sexes. In *C. testaceus*, captures were strongly male-biased (18:2), with no statistical differences (*p* = 0.06) between any treatments and the control.

## 4. Discussion

Our data represent the first test of common cerambycid pheromones, which are widely shared among related species, in South America west of the Andes [16,17,18]. The results showed that three of the eight compounds tested in Chile consistently attracted significant numbers of two native longhorn beetles, suggesting that the compounds may be pheromone components for these species. The data from other species that were not caught in statistically significant numbers may also provide clues to additional possible pheromones. In particular, the low number of replicates and the variability in trap captures between sites may have obscured differences between compounds and controls, even though the trap captures with a treatment were several times those of controls. It is also possible and even likely that the pheromones of some species may consist of blends, and so individual compounds may be only weakly attractive [29].

*Calydon submetallicum* and *E. laetus* catches in traps baited with 3-OH-6-2-Kt represented close to 60% of the total number of beetles caught. This chemical is one of the most widely shared of the known cerambycid pheromone components used by species in the subfamily Cerambycinae. In addition, the sex ratio of total captures in *C. submetallicum* and *E. laetus* were slightly female-biased (~6:4; sex ratios of the field populations are unknown), similar to reports from field trials with other cerambycine species [43,44]. However, *E. laetus* catches were male-biased in the case of 3-OH-6-2-Kt. The fact that both sexes were attracted indicates that 3-OH-6-2-Kt and the diols are likely to be part of a male-produced aggregation-sex pheromone, as occurs in almost all cerambycine species for which pheromones are known (ref. [8], but see [10] for the single exception to date). It is also noteworthy that *C. submetallicum* responded almost exclusively to 3-OH-6-2-Kt (>99% of captures for both studies), whereas *E. laetus* responded significantly to three compounds, analogous to other Cerambycinae species in similar studies where either the same or similar compounds were tested individually at a single site and time [12,45].

R*,R*- and R*,S*-diols were also significantly attractive for *E. laetus*, both showing female bias. Both compounds have been identified as pheromone components, sometimes in combination with 3-OH-6-2-Kt [6], or individually [35] in cerambycines, including Callidiini (*Calydon* tribe [40]). However, the diols have not previously been reported as attractants for the tribe Dichophyiini (*Eryphus* tribe [40], previously Heteropsini [46]). It is possible that these compounds, if they are components of the *E. laetus* pheromone but not of *C. submetallicum*, might be minor pheromonal components, and might serve for pheromone-based reproductive isolation between both species.

The subfamily Spondylininae and the tribe Monochamini have no known representatives in Chile [21,40,41,42]. This may explain why fuscumol and fuscumol acetate (known pheromones from Spondylidinae [47]) did not attract significant numbers of any Chilean species (except for male *E. laetus*). Similarly, monochamol did not significantly attract any endemic species, apart from *E. laetus* (when total catches were compared) which was caught in traps baited with all the test compounds. Even though monochamol is known only as a pheromone for species in the subfamily Lamiinae, it has been reported as an attractant for several cerambycines [9].

Even though the number of species caught was relatively limited, the attraction appeared to be at least partially correlated with phylogeny. For instance, the two cerambycine species that were caught in statistically significant numbers were generally attracted to chemicals which had been previously described from Cerambycinae in other regions, congruent with the parsimony hypothesis for pheromonal chemical structures within a particular taxon [48,49]. Thus, at least some native Chilean species may share pheromone compounds with Nearctic relatives, which can be verified in the future by collection and analysis of volatiles released by live specimens. That being said, only eleven cerambycid species (less than 7% of all described Chilean cerambycids [21]) were collected in our studies in areas where the potential for captures was much higher. For example, in Study 2, where the sampling effort was equivalent to 1512 trap-days (traps/site × sites × trapping days), we collected 538 specimens from 11 species, whereas at least twice that number of species should have been present as adults in those areas/habitats at the time of the year that the experiment was deployed. This suggests that many of the Chilean species are using pheromones other than the limited number of compounds that we tested, or that the pheromones of some species are blends [29,50]. Other authors have noted similar results when testing limited numbers of generic cerambycid pheromones or pheromone blends [23], concluding that the lures used were not able to sample the full cerambycid biodiversity in the study area. It is also likely that at least some of the compounds we tested are more frequently shared among species from the same biogeographical region (Holoarctic = Nearctic [North America] + Palearctic [Eurasia)] [28,51]). Our results are also consistent with the fact that the geographic area southwest of the Andes has some relatively unique entomofauna, resulting from geographic isolation [52]. This hypothesis is at least partially validated in our study by the absence of captures of *Proholopterus chilensis* Bl. (Cerambycinae, Proholopterini [1]), a native species which was present in high densities in the study sites in the La Araucania region in January 2019. In fact, in another field trial run parallel to Study 2 for one week at the same site in Maquehue, we captured a relatively large number of *P. chilensis* specimens when using the same type of trap baited with conspecific virgin females (TC and DA, unpublished data).

Differences in morphological characters among easily-distinguishable species are not always enough for reproductive isolation purposes in the Cerambycidae [53,54]. Thus, the attraction of two or more species to the same pheromone compound has the potential to interfere with reproductive isolation, i.e., cross-attraction to heterospecifics would be a major waste of energy and mating effort. This is particularly important in cases of synchronic or overlapping seasonal phenologies among sympatric species, such as *E. laetus* and *C. submetallicum*, which were both significantly attracted to 3-OH-6-2-Kt. Thus, reproductive isolation may occur via alternative mechanisms, such as different circadian rhythms, different host plants or microhabitats (e.g., plant structure or preferred height in the canopy), and/or, as described above, by the existence of pheromone blends that differ in proportions and/or components [11,13,55].

The phenology data from Study 2 showed that the activity periods of *E. laetus* and *C. submetallicum* flights were coincident and relatively long, lasting 8–10 weeks or more, suggesting long emergence periods or adult life cycles, or multivoltinism. Published data on adult activity from southern Chilean locations [56,57] corroborate these long activity periods, with those authors stating that *C. submetallicum* were observed between October and January. Thus, our results extended the flight period by ca. two months toward the end of the summer. The same authors reported *E. laetus* activity between September and February; thus, our results extend the activity period for several more weeks.

Considering the three most abundant species collected, all are native to western South America (Chile and Argentina [41]), all have diverse host plants (up to 21 in *E. laetus* [58]), and all are of economic importance in Chile. *Eryphus laetus* is reported as a minor pest in apple branches in southern Chile [56] and is quarantined as a potentially invasive species by the USA [59]. *Chenoderus testaceus* and *C. submetallicum* attack *Nothofagus* species [56], infesting the inner bark, but usually do not cause significant damage. However, *C. submetallicum* has been reported to cause significant damage to endemic plants in Chile [60], particularly for *Nothofagus glauca*, which is listed as vulnerable. No pheromone-based strategies have been developed in Chile against any of these species, and so the compounds found to attract these species in our studies may have a role in both monitoring and control tactics in agriculture and forestry (e.g., as in [61]). The attractants may also be useful for biological studies, such as biodiversity surveys and studies of population dynamics. However, because of the conserved status of these compounds among related taxa, and considering the uniqueness of the Chilean fauna and the potency of these attractants, care should be taken to avoid potential negative effects from using them extensively and intensively, in case of harm to beneficial, rare, or endangered species [23,24].

## 5. Conclusions

3-Hydroxy-2-hexanone attracted significant numbers of *Calydon submetallicum* and *Eryphus laetus*. However, (2R*,3S*)- and (2R*,3R*)-2,3-hexanediol only significantly attracted *E. laetus*. Males and females of *C. submetallicum* and *E. laetus* were both attracted to 3-hydroxy-2-hexanone, (2R*,3S*)-, and (2R*,3R*)-2,3-hexanediol, indicating that the chemicals are likely aggregation-sex pheromone components. Our data suggest that some Chilean cerambycid species share pheromone components with related species from other continents, further substantiating that these compounds have been conserved within the family through evolutionary time.

## Figures and Tables

**Table 1:** Study 1, mean/day (±SEM), T^2^- and *p*-value, and total catches of cerambycids by compounds tested for 5 days, January 2011, Ñuble and Los Rios regions, Chile.

Species	Treatments									Total
	3-OH-6-2-Kt	R*S*- Diol	R*R*- Diol	Monocha- mol	Fuscumol	Fuscumol Acetate	Control	T^2^	*p*	
*E. laetus*	0.35 ± 0.29	0.50 ± 0.50	0.10 ± 0.10	0.05 ± 0.05	0	0.10 ± 0.10	0.05 ± 0.05			23
*C. submetallicum*	0.80 ± 0.22 a ^1^	0 b	0 b	0 b	0 b	0 b	0 b	1 × 10^30^	<0.0001	16
*H. corralensis*	0	0	0	0.05 ± 0.05	0	0	0			1

^1^: different small letters in *C. submetallicum* row indicate significant differences between compounds (Friedman and Conover tests, α = 0.05). * is a chemistry nomenclature notation for relative configuration.

**Table 2:** Study 2, mean/day (±SEM), T^2^- and *p*-values, and total catches of cerambycids by compounds tested for 56 days, January–March 2019, La Araucania region, Chile.

Species	Treatments											Total
	3-OH-6-2-Kt	R*S*-diol	R*R*-diol	Monochamol	Fuscumol	Fuscumol acetate	2-Methyl	Geranyl	Control	T^2^	*p*	
							Butanol	Acetone				
*E. laetus*	0.625 ± 0.358 a ^1^	0.304 ± 0.241 abc	0.220 ± 0.167 abcd	0.161 ± 0.109 cde	0.089 ± 0.063 defg	0.089 ± 0.031 bcde	0.012 ± 0.006 g	0.029 ± 0.012 efg	0.071 ± 0.063 fg	7.3	0.0004	269
*C. submetallicum*	1.286 ± 0.537 a	0 b	0 b	0 b	0 b	0.006 ± 0.006 b	0.006 ± 0.006 b	0 b	0 b	7.8	0.0003	218
*C. testaceus*	0.024 ± 0.006	0.024 ± 0.006	0	0.024 ± 0.006	0.029 ± 0.029	0.012 ± 0.012	0	0	0.006 ± 0.006	2.4	0.0645	20
*H. corralensis*	0.006 ± 0.006	0.012 ± 0.012	0	0	0.006 ± 0.006	0.012 ± 0.012	0	0.029 ± 0.029	0			11
*X. flavonitida*	0.006 ± 0.006	0.006 ± 0.006	0.006 ± 0.006	0	0.012 ± 0.012	0.006 ± 0.006	0	0.012 ± 0.012	0			8
*A. cristatipennis*	0	0	0	0	0.006 ± 0.006	0.006 ± 0.006	0	0.006 ± 0.006	0.006 ± 0.006			4
*N. humeralis*	0	0.006 ± 0.006	0	0.006 ± 0.006	0	0	0	0	0.006 ± 0.006			3
*M. quadrimaculatus*	0	0.006 ± 0.006	0	0	0	0.006 ± 0.006	0	0	0			2
*C. sulcicorne*	0	0	0	0	0	0	0.006 ± 0.006	0	0			1
*X. semipolita*	0	0	0	0	0	0.006 ± 0.006	0	0	0			1
*S. virescens*	0	0	0	0	0	0.006 ± 0.006	0	0	0			1

^1^: different small letters in a row (species) indicate significant differences between compounds (Friedman and Conover tests, α = 0.05). * is a chemistry nomenclature notation for relative configuration.

**Table 3:** Study 2, mean/day (±SEM), T^2^- and *p*-values, and total catches for *E. laetus*, *C. submetallicum,* and *C. testaceus* males and females, in traps baited with test pheromones over 56 days, January–March 2019, La Araucania region, Chile.

	*E. laetus*		*C. submetallicum*		*C. testaceus*	
Treatments	Males	Females	Males	Females	Males	Females
3-OH-6-2-Kt	0.345 ± 0.221 a ^1^	0.280 ± 0.137 a	0.554 ± 0.206 a	0.732 ± 0.331 a	0.024 ± 0.010	0
R*S*-diol	0.089 ± 0.071 abcd	0.214 ± 0.170 abcd	0 b	0 b	0.024 ± 0.027	0
R*R*-diol	0.089 ± 0.071 abcd	0.131 ± 0.096 abcde	0 b	0 b	0	0
Monochamol	0.065 ± 0.049 cde	0.095 ± 0.060 bcdef	0 b	0 b	0.024 ± 0.027	0
Fuscumol	0.048 ± 0.039 defg	0.042 ± 0.024 efg	0 b	0 b	0.024 ± 0.041	0.006 ± 0.006
Fuscumol acetate	0.030 ± 0.012 bcd	0.060 ± 0.021 cdef	0.006 ± 0.006 b	0 b	0.024 ± 0.021	0
2-Methylbutanol	0 g	0.012 ± 0.006 g	0.006 ± 0.006 b	0 b	0	0
Geranylacetone	0 g	0.030 ± 0.012 defg	0 b	0 b	0	0
Control	0.024 ± 0.024 efg	0.048 ± 0.039 fg	0 b	0 b	0	0.006 ± 0.006
**T^2^**	6.89	4.47	6.2	1 × 10^30^	2.46	
***p***	0.0006	0.0053	0.001	<0.0001	0.0599	
**Total**	116	153	95	123	18	2

^1^: different small letters in a column (species × sex) indicate significant differences between compounds (Friedman and Conover tests, α = 0.05). * is a chemistry nomenclature notation for relative configuration.

## Data Availability

The data presented in this study are available on request from the corresponding author.
